# Possible causes of experiencing problems with sick leave questions in telephone nursing

**DOI:** 10.1080/03009734.2017.1385665

**Published:** 2017-10-18

**Authors:** Linda Lännerström, Inger K. Holmström, Kurt Svärdsudd, Thorne Wallman

**Affiliations:** a Uppsala University, Department of Public Health and Caring Sciences, Family Medicine and Preventive Medicine Section, Uppsala, Sweden; b Uppsala University, Centre for Clinical Research Sörmland, Eskilstuna, Sweden; c Mälardalen University, School of Health, Care and Social Welfare, Västerås, Sweden; d Uppsala University, Department of Public Health and Caring Sciences, Health Services Research Section, Uppsala, Sweden

**Keywords:** Nursing, primary health care, sickness certification, sick leave, sick-listing, social insurance medicine, telephone nursing

## Abstract

**Background:**

Registered nurses at primary health care centres in Sweden receive about 20 million telephone calls annually. Questions related to sick leave occur regularly. Previous studies conclude that those calls often are perceived as problematic. The aim of this study was to explore factors associated with problems regarding sick leave questions in telephone nursing.

**Methods:**

A questionnaire was distributed to all registered nurses (*n* = 185) working with telephone nursing in 26 Swedish primary health care centres, of whom 114 (61.6%) responded. Based on the results of a Spearman correlation analysis a logistic regression analysis was performed of significant exposure variables on outcome (perceived problems).

**Results:**

Significant exposure variables were: experience of telephone nursing, age, being educated in social insurance medicine, and frequency of telephone calls with sick leave questions. Young age was associated with more problems than old age. Those having education in social insurance medicine reported fewer problems than those who had not, and so did those having few telephone calls with sick leave questions as compared with those who had many.

**Conclusions:**

Young age, lack of education in insurance medicine, and high frequency of sick leave questions increased the perceived problem level in telephone nursing.

## Introduction

Registered nurses working at Swedish primary health care centres receive about 20 million telephone calls annually (1) during telephone nursing when collecting information, assessing health care needs, giving advice, support, and education, and helping callers to the right level of care (2–5). Sick leave questions, here defined as questions related to sick leave, sickness certification, or social insurance rules, are among the issues telephone nurses handle on a regular basis. In a previous study 92% of the registered nurses described having such calls every week (6). The outcomes of the calls are often to make physician appointments but also to guide, advise, and co-ordinate for callers (6–8), thus the role of registered nurses when working with sick leave consists of different parts depending on the callers’ questions.

The handling of telephone calls with sick leave questions has been described in two previous Swedish studies (6,7) as problematic to manage. In a previous study from the present project (6) the majority of registered nurses (64.9%) experienced problems when handling telephone calls with sick leave questions weekly or more often. Problems reported dealt with what type of information to give (93.9%), where to refer the patient (89.5%), and how to handle dissatisfied patients (93.9%).

The underlying factors causing the registered nurses to assess sick leave questions as problematic or not problematic are so far unknown. The aim of this study was to explore possible underlying factors associated with registered nurses experiencing problems with sick leave questions in telephone nursing.

## Materials and methods

### Study population

All registered nurses working with telephone nursing at 26 primary health care centres in mid-Sweden were invited to participate. Of the 26 primary health care centres, 20 agreed to participate in the study and to contact their registered nurses (*n* = 185). The primary health care centres provided a contact person who was informed about the study and in turn informed the registered nurses about design of the study, checked informed consent, and distributed the questionnaire.

### Data collection

A questionnaire with 120 questions was distributed to the 185 registered nurses (6). The questionnaire was developed from a previous questionnaire repeatedly used among physicians (9,10), and some of the questions were constructed from findings in a previous focus group study (7). The questionnaire was tested on six telephone nurses, and after this procedure some minor changes were made to clarify definitions of concepts and to improve layout. The final questionnaire contained questions about the work with sick leave questions, problems, knowledge, and demographic questions. The Regional Research Ethics Board, Uppsala, Sweden, approved the study (Dnr. 2014/156).

### Statistical considerations

Data were analysed with the SAS software package, version 9.3 (11). Out of the 185 registered nurses in the sample 114 (61.6%) responded. Of 71 non-participants 12 were from private primary health care centres and 59 from public. There were no other characteristics available for the non-participants except for the ownership of their workplaces. Among the responders on average 0.6% of the data in the questionnaire were missing.

The outcome variable was the response to the question ‘How often do you find it problematic to handle sick leave questions?’ The possible responses were ‘never’ (*n* = 5), ‘a few times a year’ (*n* = 2), ‘a few times a month’ (*n* = 32), ‘1–5 times a week’ (*n* = 61), ‘6–10 times a week’ (*n* = 14), and ‘more than 10 times a week’ (*n* = 0). The variable was dichotomized into ‘less than once a week’ (problem = 0) and ‘once a week or more often’ (problem = 1).

The first 14 questions in the questionnaire were chosen as exposure variables: ‘age’, ‘sex’, ‘having a specialist nurse examination’, ‘total time working with telephone nursing’, ‘working in publicly or privately operated primary health care centre’, ‘working part-time or full-time’, ‘been sick-listed for more than seven days during the last five years’, ‘has a workplace policy for handling of sick-listings’, ‘sick-listing is a work environmental problem’, ‘supported by the nearest manager regarding sick leave questions’, ‘I have a role in the care of sick-listed patients’, ‘I am educated in social insurance medicine’, and ‘my frequency of telephone calls with sick leave questions’. The remaining questions dealt with other aspects of telephone consultations.

First, a univariate analysis of differences between groups was performed with Student’s *t* test for continuous variables and the chi-square test for discrete variables. Based on these results a logistic regression analysis was performed with the outcome (problem) as the dependent variable and the significant exposure variables as independent variables. To check the results of the correlation analysis a logistic regression was also performed with all the 14 exposure variables, with backward elimination and with stepwise inclusion of exposure variables. The results of all these analyses yielded almost identical results.

**Figure 1.**
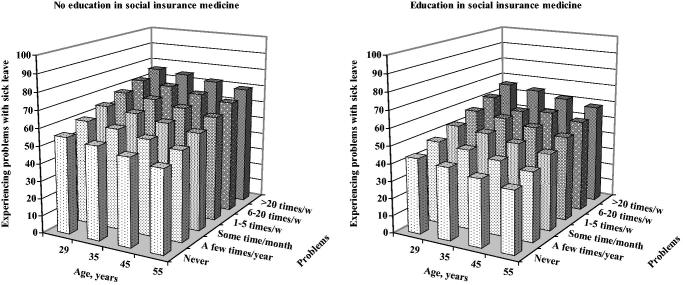
Association between age, frequency of patients asking sick leave questions, and experienced problems with sick leave questions among registered nurses with no education in social insurance medicine, and registered nurses with such education.

A sensitivity analysis was performed with the outcome variable trichotomized into ‘less than weekly’ (*n* = 34), ‘1–5 times a week’ (*n* = 61), and ‘6 times a week or more often’ (*n* = 14). Data for Figure 1 were obtained from the logistic analysis model. A *P* value of less than 0.05 was regarded as statistically significant.

## Results

### Characteristics of the study population

The characteristics of the study population subdivided into those who reported small problems with sick listing and those who reported large problems are shown in Table 1. Those who reported small problems were older (*P* < 0.0005), had a longer experience of telephone nursing (*P* < 0.005), had more education in social insurance medicine (*P* < 0.05), and had less frequent telephone calls with problematic sick leave questions (*P* < 0.001). A sensitivity analysis was performed with the outcome classified into three groups (Table 2). The results were basically the same with the exceptions that ‘Has a specialist nurse examination’ now became significant between the groups, while ‘Educated in social insurance medicine’ did not.

**Table 1.** Characteristics of the study population.

	No problems^a^		Problems^b^		
	*n*	Mean (SD) or %	*n*	Mean (SD) or %	*P*
*n*	39		75		
Age		53.4 (8.3)		46.6 (10.3)	<0.0005
Women, %	39	100.0	73	96.0	n.s.
Has a specialist nurse examination, %	31	79.5	51	68.0	n.s.
Has worked with telephone nursing 6 years or more, %	29	74.4	37	49.3	<0.005
Working in County Council-operated primary health care centre, %	29	74.4	63	84.0	n.s.
Working full time, %	24	61.5	46	61.3	n.s.
Telephone nursing <50% of working hours, %	15	38.5	29	38.7	n.s.
Own sick-listing <7 days during last 5 years, %	15	38.5	42	56.0	n.s.
Workplace policy for handling sick leave	25	67.6	47	42.0	n.s.
Handling sick leave questions is for me no work environmental problem, %	19	48.7	31	41.3	n.s.
Gets no support from managers, %	4	11.4	10	13.5	n.s.
Has a role in health care of sick-listed patients, %	28	71.8	50	67.6	n.s.
Educated in social insurance medicine, %	4	10.3	1	1.4	<0.05
Telephone calls with sick leave questions once a week or more often, %	32	82.0	73	97.3	<0.001

a No problems = problematic telephone nursing calls less than once a week.

b Problems = problematic telephone nursing calls once a week or more often.

**Table 2.** Characteristics of the study population with outcome classified in three groups.

	Problem group 0^a^		Problem group 1^b^		Problem group 2^c^		
	*n*	Mean (SD) or %	*n*	Mean (SD) or %	*n*	Mean (SD) or %	*P*
*n*	34		61		14		
Age		53.6 (8.6)		47.8 (10.0)		41.4 (10.3)	<0.0001
Women, %	34	100.0	61	96.7	14	92.9	n.s.
Has a specialist nurse examination, %	34	85.3	61	72.1	14	50.0	<0.05
Has worked with telephone nursing 6 years or more, %	26	76.5	34	55.7	3	21.4	<0.0001
Working in County Council-operated primary health care centre, %	26	76.5	52	85.3	11	78.6	n.s.
Working full time, %	20	58.8	35	57.4	11	78.6	n.s.
Telephone nursing <50% of working hours, %	13	38.2	26	42.6	3	21.4	n.s.
Own sick-listing >7 days during last 5 years, %	13	38.2	34	55.7	8	57.1	n.s.
Workplace policy for handling sick leave	22	66.7	40	65.6	7	50.0	n.s.
Handling sick leave questions is for me no work environmental problem, %	14	41.2	27	44.3	4	28.6	n.s.
Gets no support from managers, %	4	12.1	8	13.3	2	14.3	n.s.
Has a role in health care of sick-listed patients, %	25	73.5	42	68.9	8	61.5	n.s.
Educated in social insurance medicine, %	3	8.8	1	1.7	0	0	n.s.
Telephone calls with sick leave questions once a week or more often, %	28	82.4	59	96.7	14	100.0	<0.0001

a Problem group 0 = problematic telephone nursing calls less than onsce a week.

b Problem group 1 = problematic telephone nursing calls 1–5 times a week.

c Problem group 2 = problematic telephone nursing calls 6 times a week or more often.

A logistic regression was performed with outcome as the independent variable and age, total time working with telephone nursing, education in social insurance medicine, and frequency of sick leave questions as independent variables. Total time working with telephone nursing at primary health care centres became non-significant, due to a strong correlation to age.

Age was negatively related to outcome, i.e. older registered nurses reported fewer problems than younger ones did (Table 3). Education in social insurance medicine was also inversely related to outcome. Those who had such education reported fewer problems than those without. Finally, those who frequently encountered patients with sick leave questions reported more problems than those who encountered fewer such patients. Wald’s χ indicates the impact of the exposure variables on outcome. Frequency of sick leave questions had the largest impact, followed by age and education in social insurance medicine.

**Table 3.** Logistic regression of age, telephone nursing experience, and social insurance medicine education on frequency of sick leave problems.

Parameter	Estimate (SE)	Wald’s χ	HR	95% CI	*P*
Age	−0.0670 (0.0241)	7.7	0.94	0.89–0.98	=0.005
Educated in social insurance medicine	−2.4884 (1.2188)	4.2	0.08	0.008–0.91	<0.05
Frequency of sick leave questions	0.9309 (0.3081)	9.1	2.54	1.39–4.64	<0.005

Among those with no education in social insurance medicine the lowest level of experienced problems with sick leave questions was found in the oldest age group with few patients asking sick leave questions (47.4%), and the highest level among the youngest with the highest frequency of sick leave questions (75.8%) (Figure 1). The corresponding proportions among those with social insurance medicine education were 35.7% and 65.8%.

## Discussion

Significant factors affecting experienced problems with sick leave questions in telephone nursing were age, education in social insurance medicine, and frequency of telephone calls with sick leave questions. Young registered nurses experienced more problems than the older nurses did. Registered nurses educated in social insurance medicine reported fewer problems than those without such education, as did the registered nurses having fewer telephone calls with sick leave questions. Of the three significantly associated factors, frequency of sick leave questions had the largest impact on experiencing problems, followed by age and education in social insurance medicine.

The finding that the registered nurses’ age was associated with fewer experienced problematic sick leave questions is most probably linked to the fact that skills and knowledge increase with age (12). Similar findings have been described among physicians (13). It is no surprise that registered nurses with many sick leave questions telephone calls more often found sick leave questions problematic since more exposure to sick leave questions should increase the risk of encountering problems.

Hypothetically, encountered problems might be reduced by intervention towards the underlying factors. Of these, education in social insurance medicine is a possible way of developing more competence and thereby reducing the perceived problem level. A reduction of registered nurses’ experienced problem levels by education was previously proposed in two interview studies with registered nurses (7,8) and is furthermore suggested by the results in the present study. That registered nurses, and also physicians, lack and express a large need for education in social insurance medicine has been described in several studies (6,7,9,14,15). Thus, this could be a target for further research.

The strengths of the study were that the sample included a fairly large population, although a substantial proportion of the registered nurses declined the invitation to the study. It is possible that there is a selection bias among the participating registered nurses in terms of being more positive towards the subject and therefore responding positively to the invitation. The use of contact persons informing participants about the study could have affected the rate of participation depending on the engagement of the contact person. The analysis model was robust in the sense that classification of the outcome into two or three groups yielded similar results.

As described before it was not possible to carry out a non-participant analysis due to not having data on the participants declining. How the participants interpreted the questions is—as in all surveys—unknown, especially concerning concepts connected to a topic where the participants express a large lack of knowledge.

In conclusion, young age, lack of education in insurance medicine, and high frequency of sick leave questions from calling patients increased the perceived problem level in telephone nursing.
